# Primary Cutaneous Blastomycosis: An Ulcerated Wound in an Immunocompetent Patient

**DOI:** 10.7759/cureus.45786

**Published:** 2023-09-22

**Authors:** Matthew Melton, Katherine Baquerizo Nole

**Affiliations:** 1 Dermatology, University of Kentucky, Lexington, USA; 2 Dermatology, University of Cincinnati College of Medicine, Cincinnati, USA

**Keywords:** nonhealing wound, blastomyces dermatidis, adenocarcinoma, dermatology, skin blastomycosis

## Abstract

The report describes a case of cutaneous blastomycosis. The patient was a healthy elderly male with a history of rectal adenocarcinoma, who presented with an ulcerating wound on his left buttock. Fungal culture was positive for Blastomyces dermatitidis, and the patient was treated with itraconazole followed by voriconazole for three months, which led to clinical resolution of the infection.

This highlights an atypical case of blastomycosis, which presented as an isolated skin condition, without pulmonary or hematogenous complications. There are many challenges to diagnosing blastomycosis due to its wide range of symptoms, which can mimic other conditions, making it difficult to diagnose. Accurate diagnosis of blastomycosis is crucial to provide effective treatment and prevent potential complications, such as the infection spreading to other parts of the body and causing systemic symptoms. The report concludes by emphasizing the importance of a high index of suspicion for the diagnosis of cutaneous blastomycosis.

## Introduction

Blastomycosis is a rare systemic fungal infection, most commonly caused by the inhalation of the spores of the Blastomyces dermatitidis fungus. The disease is commonly found in regions surrounding the Great Lakes, as well as the Mississippi and St. Lawrence River valleys in North America. The most common manifestation of Blastomycosis infection is a pulmonary infection, most often in immunocompromised individuals, with other constitutional symptoms. Cutaneous blastomycosis is another manifestation of blastomycosis; however, it is rarely the only manifestation of infection. The rarity of an isolated cutaneous infection with blastomycosis is significant as it underscores the importance of considering blastomycosis as a potential causative agent for various skin lesions. Here, we describe a case of isolated cutaneous blastomycosis that presented in an immunocompetent individual.

## Case presentation

The patient was a male in his 60s living in rural Ohio, with no recent out-of-state travel. He presented to dermatology due to an ulcerated wound on the left buttock. He had a past medical history significant for rectal adenocarcinoma Stage 3b status post sigmoid and rectum resection and chemoradiation one year prior. Notably, he was not immunocompromised at the time, based on comprehensive metabolic panel (CMP) and complete blood count (CBC) with differential within normal limits, and had not undergone chemotherapy within the past year. The patient did take a weekly testosterone injection for hypotestosteronism. Apart from this, he had no chronic conditions and tested negative for HIV during the workup.

**Figure 1:**
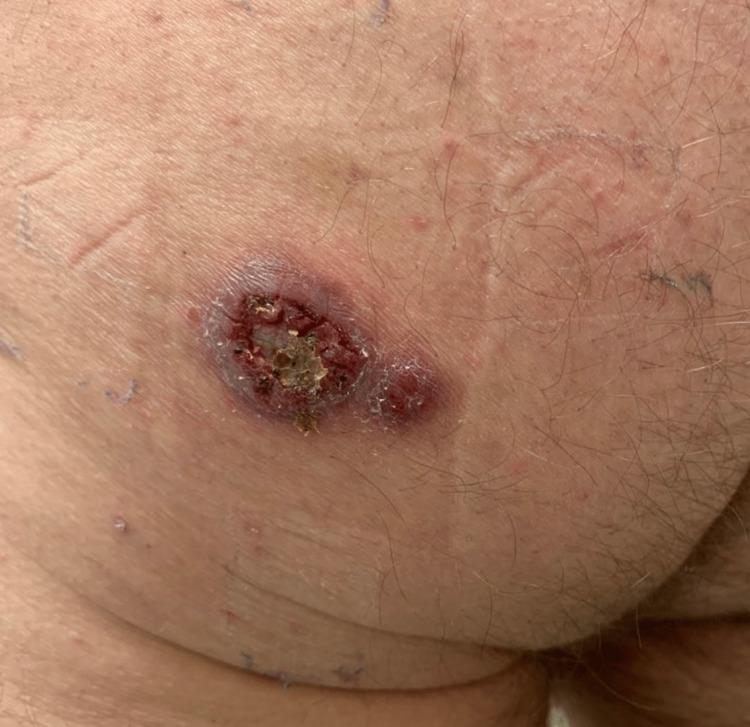
Initial presentation A 2cm violaceous ulcerated nodule with hemorrhagic crusting and surrounding scale with a 0.5cm violaceous satellite nodule on the left buttock.

The lesion started as a 3mm papule over the lower left buttock. Over the course of one month, the lesion progressed to ulceration despite attempts at manual drainage. He received a course of sulfamethoxazole/trimethoprim and doxycycline from his primary care physician, without improvement. Routine culture was collected and was negative for bacterial growth. The location of the lesion correlated with the testosterone injection site. He worked in roofing and reported tearing down a drywall at home two months prior. He did report having an outdoor shed at home. Upon physical examination, a 2cm violaceous ulcerated nodule with a 0.5cm satellite nodule was observed on the left buttock (Figure 1). The lesion was asymptomatic and the patient reported that it stained his clothing with a light-yellow fluid.

**Figure 2:**
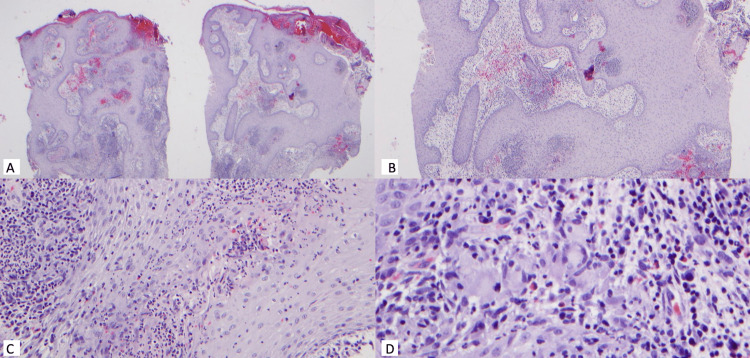
H&E staining of biopsy H&E staining showing neutrophilic and hemorrhagic crusting, marked pseudoepitheliomatous hyperplasia, many neutrophilic abscesses, superficial dermal edema and a dense, diffuse mixed infiltrate of lymphocytes, neutrophils, plasma cells, histiocytes and several giant cells throughout the superficial dermis. Original magnification 40x (A), 100x (B), 200x (C), 400x (D).

Two punch biopsies were collected and sent for pathology and tissue culture, routine, anaerobic, fungal, and mycobacterial. H&E staining showed neutrophilic and hemorrhagic crusting, marked pseudoepitheliomatous hyperplasia, many neutrophilic abscesses, superficial dermal edema and a dense, diffuse mixed infiltrate of lymphocytes, neutrophils, plasma cells, histiocytes and several giant cells throughout the superficial dermis (Figure 2). Period acid-Shiff plus diastase and acid-fast stains for microorganisms were negative. Fungal culture was positive for Blastomyces dermatitidis. Aerobic, anaerobic, and mycobacterial cultures were negative. Blastomyces urine and serum antigen were negative.

**Figure 3:**
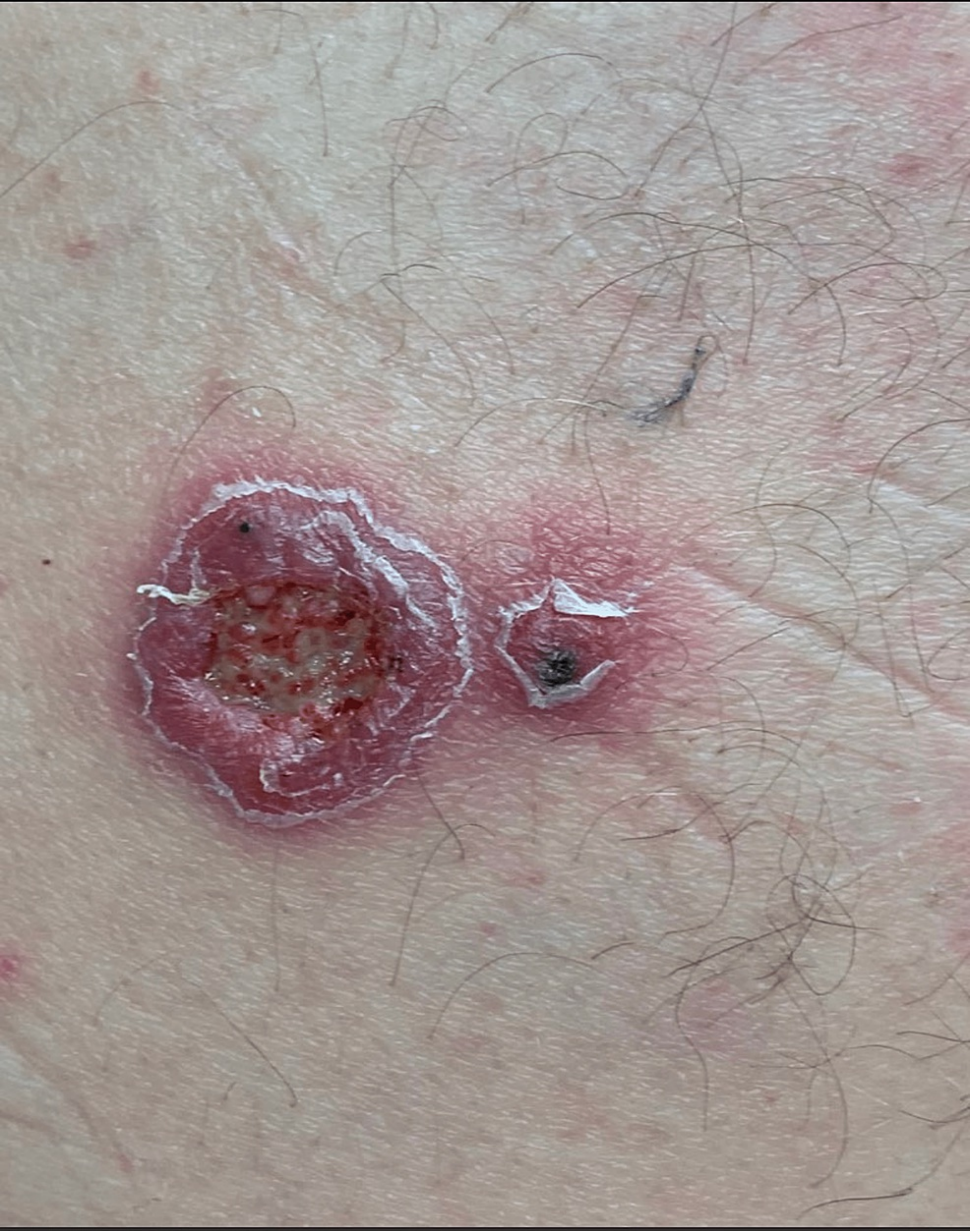
Follow-up presentation after six weeks of treatment Follow-up presentation after six weeks of treatment with itraconazole. A 1.5cm violaceous ulcerated nodule with granulomatous tissue formation with a 0.5cm satellite nodule with central necrosis on the left buttock.

**Figure 4:**
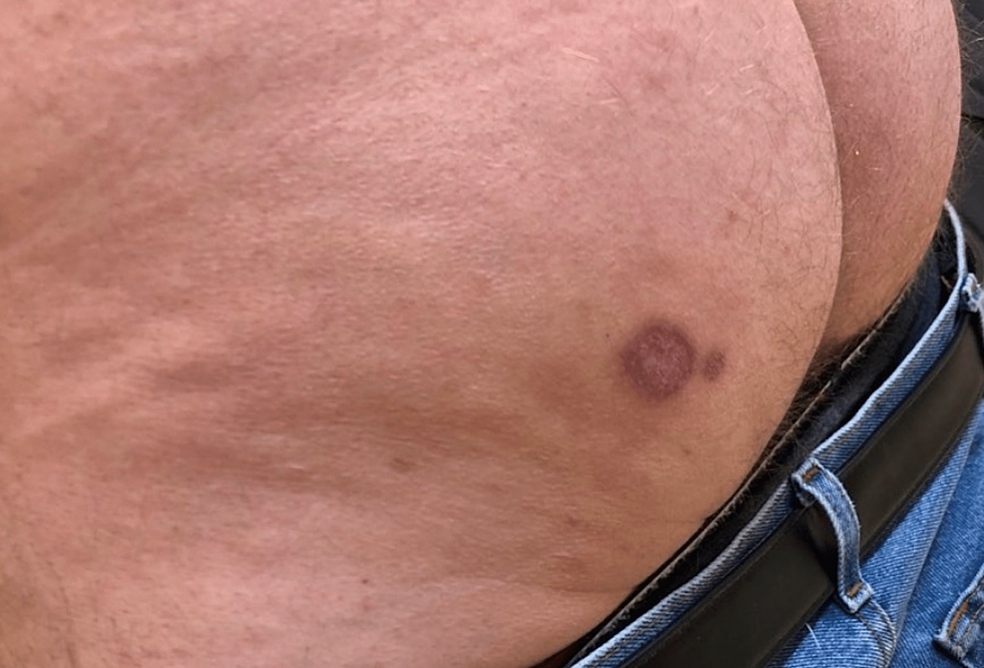
Four-month follow-up Near complete resolution of the prior wound four months after initial presentation. A 1.2cm maroon macule with a 2-3mm satellite macule.

Based on results, the patient was medically evaluated for long-term antifungal therapy, including baseline hematologic, renal, and hepatic function assessments. The patient was then treated with itraconazole 200mg three times daily for three days, then 200mg twice daily for approximately six weeks, at which point it was discontinued due to development of lower extremity edema. At this point, the patient was beginning to have clinical improvement, showing a 1.5cm violaceous ulcerated nodule with granulomatous tissue formation with a 0.5cm satellite nodule with central necrosis on the left buttock (Figure 3). He was started on voriconazole 200mg twice daily for three additional months. After three months, the patient experienced joint stiffness and hand edema, leading to the discontinuation of voriconazole due to clinical resolution (Figure 4).

At a two-year clinical follow-up, there was no evidence of recurrence. The patient was able to return to daily activities within the first month of treatment. Lower extremity edema and joint pain subsided with removal of voriconazole.

## Discussion

Blastomyces dermatitidis is endemic to the Mississippi and Ohio river basins, encompassing many Eastern and Southern states in the United States [1]. This is important, as the patient was from this region. Primary cutaneous blastomycosis infections are a rare presentation of blastomycosis, affecting only the skin and subcutaneous tissues. Cutaneous blastomycosis may present as one or more verrucous lesions that are violet-to-grey in color or as an ulcerated lesion with heaped-up borders [2]. Additionally, cutaneous blastomycosis commonly has microabscesses and satellite lesions in the periphery [2]. The differential diagnosis of blastomycosis is broad, encompassing squamous cell carcinoma, pyoderma gangrenosum, granulomatous diseases such as syphilis or tuberculosis, and other fungal infections like Histoplasma capsulatum or Coccidioidomycosis [3]. In our patient the main differential diagnoses were neoplastic, including metastatic adenocarcinoma, and infectious. Histological evaluation of tissue infected with blastomycosis may present as pseudoepitheliomatous hyperplasia, hyperkeratosis, acanthosis, and hypergranulosis with neutrophilic infiltrate in the dermis or noncaseating granulomas [2]. These findings can be mistaken for pyoderma gangrenosum or granulomatous skin diseases; therefore, fungal culture is crucial for diagnosing cutaneous blastomycosis. Fungal culture of blastomycosis reveals classic round and nonencapsulated broad budding yeasts, often located within and surrounding cellular tissue [2]. Pathologic staining with Gomori methenamine silver (GMS) and periodic acid-Schiff can be used to stain Blastomyces dermatitidis. Many of these diagnostic factors matched with our patient, supported by a fungal culture with growth of Blastomyces dermatitidis.

Accurate diagnosis of this condition is crucial in order to provide effective treatment and prevent potential complications. Diagnosing blastomycosis can be challenging, as its clinical features can be similar to those of other skin conditions such as pyoderma, cellulitis, and squamous cell carcinoma [3]. In addition, skin biopsy results may not always yield a definitive diagnosis, as the fungus can be difficult to culture [4]. Therefore, a high index of suspicion is required in order to make an accurate diagnosis. Missing a diagnosis of cutaneous blastomycosis can have serious consequences. If left untreated, the infection can spread to other parts of the body and cause systemic symptoms such as fever, weight loss, and respiratory distress. In severe cases, it can lead to death.

It is also important to assess the patient’s risk factors for becoming infected with blastomycosis, such as exposure to soil, wood, or organic debris contaminated with the fungus [3]. Unfortunately, the testosterone vial was not able to be tested, and the lot number was not available, but inoculation through contaminated needles was a possibility. Risk factors for becoming infected with cutaneous blastomycosis include being immunocompromised, living or working in an area with a high incidence of the disease, and having a history of skin trauma [3]. Individuals who engage in outdoor activities such as gardening, hunting, and camping may also be at increased risk.

Treatment recommendations are dependent upon the location and severity of infection, host immunity status, and pregnancy. All individuals diagnosed with Blastomycosis require antifungal therapy [5]. Baseline laboratory evaluation should be conducted before initiating therapy, including assessments of hematologic, renal, and hepatic function. According to McBride, mild cutaneous disease can be treated with oral itraconazole 200mg three times daily for three days, followed by once or twice daily dosing for six to 12 months [2]. Moderate to severe disease should be treated using a lipid formulation of amphotericin B 3-5mg/kg daily or amphotericin deoxycholate 0.7-1 mg/kg daily for one to two weeks or until improvement is noted [5].

## Conclusions

This case report details a unique case of Blastomyces dermatitidis as an isolated skin infection in a healthy male. Although there are multiple reports in the literature about isolated cutaneous blastomycosis, this case presented atypically in a healthy individual. As illustrated by this case, the importance of considering atypical fungal infections when dealing with cutaneous infections cannot be understated.
